# Statistical sensitivity estimates for oscillating electric dipole moment measurements in storage rings

**DOI:** 10.1140/epjc/s10052-020-7664-9

**Published:** 2020-02-10

**Authors:** J. Pretz, S. P. Chang, V. Hejny, S. Karanth, S. Park, Y. Semertzidis, E. Stephenson, H. Ströher

**Affiliations:** 1 Institut für Kernphysik, Forschungszentrum Jülich, 52425 Jülich, Germany; 2 III. Physikalisches Institut B, RWTH Aachen University, 52056 Aachen, Germany; 3 JARA-FAME, Forschungszentrum Jülich, RWTH Aachen University, Aachen, Germany; 4 Center for Axion and Precision Physics Research, IBS, Daejeon, 34051 Republic of Korea; 5 Department of Physics, KAIST, Daejeon, 34141 Republic of Korea; 6 Marian Smoluchowski Institute of Physics, Jagiellionian Univsersity, Cracow, Poland; 7 Indiana Univ., Bloomington, IN 47408 USA; 8 JARA–FAME (Forces and Matter Experiments), Forschungszentrum Jülich, RWTH Aachen University, Aachen, Germany

## Abstract

In this paper analytical expressions are derived to describe the spin motion of a particle in magnetic and electric fields in the presence of an axion field causing an oscillating electric dipole moment (EDM). These equations are used to estimate statistical sensitivities for axion searches at storage rings. The estimates obtained from the analytic expressions are compared to numerical estimates from simulations in Chang et al. (Phys Rev D 99(8):083002, 2019). A good agreement is found.

## Introduction and motivation

Axions and axion like particles (ALPs) are candidates for dark matter. There is thus a huge experimental effort for the search of these kind of particles. For a detailed review, we refer the reader to references [2, 3]. Axions and ALPs can interact with ordinary matter in various ways. Reference [4] identifies three terms:

1 $$\begin{aligned} \frac{a}{f_0} \, F_{\mu \nu } {\tilde{F}}_{\mu \nu }, \quad \frac{a}{f_a} \, G_{\mu \nu } {\tilde{G}}_{\mu \nu }, \quad \frac{\partial _\mu a}{f_a} {\bar{\varPsi }}_f \gamma ^\mu \gamma _5 \varPsi \end{aligned}$$

describing the coupling to photons, gluons and to the spin of fermions, respectively. The vast majority of experiments makes use of the first term [e.g. Cavity experiments (ADMX), helioscopes (CAST), light-through-wall experiments (ALPS)]. In addition, astrophysical observations also provide sensitive limits to the axion-photon coupling. In general, it is rather difficult for these experiments to reach masses below $10^{-6}\,{\mathrm{eV}}$, one reason being that the axion wave length becomes too large. Furthermore, these experiments are measuring rates proportional to at least a small amplitude squared.

For the second (and third) term in the list (1) this is different. It turns out that the second term has the same structure as the QCD-$\theta$ term which is also responsible for an electric dipole moment (EDM) of nucleons. The axion field gives rise to an effective time-dependent $\theta$-term and oscillates at a frequency proportional to the mass of the axion $m_a$. This gives rise to an oscillating EDM. New opportunities to search for axions/ALPs with much higher sensitivity arise, because the signal is proportional to an amplitude *A* and not to its square. To date, NMR based methods are being used to look at oscillating EDMs [5].

Another possibility is to search for axions/ALPs in storage rings. Storage ring experiments have been proposed to search for electric dipole moments of charge particles [6, 7]. These experiments allow also, with small modifications, to search for oscillating EDMs. This possibility is discussed in this paper. Section 2 explains the principle of the experiment, how the (oscillating) EDM alters the spin motion in electromagnetic fields and leads to a polarization observable. In Sect. 3 statistical sensitivities for oscillating EDMs based on these polarization observables are presented.

## Spin motion in storage rings

The spin motion relative to the momentum vector in electric and magnetic fields is governed by the Thomas-BMT equation [8–10]:

2 $$\begin{aligned} \frac{d \mathbf {S}}{dt}= & {} (\varvec{\Omega }_{\mathrm {MDM}} + \varvec{\Omega }_{\mathrm {EDM}}) \times \mathbf {S}, \end{aligned}$$

3 $$\begin{aligned} \varvec{\Omega }_{\mathrm {MDM}}= & {} -\frac{q}{m} ~ \left[ G \mathbf {B} - \left( G-\frac{1}{\gamma ^2-1} \right) \frac{\varvec{\beta } \times \mathbf {E}}{c}\right] , \end{aligned}$$

4 $$\begin{aligned} \varvec{\Omega }_{\mathrm {EDM}}= & {} -\frac{\eta q}{2 m c} \left[ \mathbf {E} + c \varvec{\beta } \times \mathbf {B} \right] . \end{aligned}$$

$\mathbf {S}$ in this equation denotes the spin vector in the particle rest frame, *t* the time in the laboratory system, $\beta$ and $\gamma$ the relativistic Lorentz factors, and $\mathbf {B}$ and $\mathbf {E}$ the magnetic and electric fields in the laboratory system, respectively. The magnetic dipole moment $\varvec{\mu }$ and electric dipole moment $\mathbf {d}$ both pointing in the direction of the particle’s spin $\mathbf {S}$ are related to the dimensionless quantities *G* (magnetic anomaly) and $\eta$ in Eq. 2:

5 $$\begin{aligned} \varvec{\mu } = g \frac{q \hbar }{2 m} \mathbf {S} = (1+G) \frac{q \hbar }{m} \mathbf {S}\, \quad \text{ and } \quad \mathbf {d} = \eta \frac{q \hbar }{2 m c} \mathbf {S}. \end{aligned}$$

**Fig. 1:**
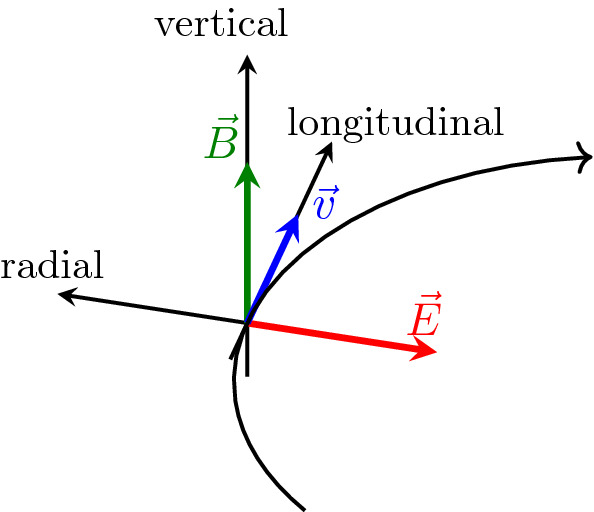
Illustration of the coordinate system used

We assume a vertical magnetic field and a radial electric field, constant in time, forcing the particle on a circular orbit. The three vectors $\mathbf {B}$, $\mathbf {E}$ and $\mathbf {v} = {\varvec{\beta }} c$ are thus mutually orthogonal, as indicated in Fig. 1. In this case

6 $$\begin{aligned} {\varvec{\Omega }}_{\mathrm {MDM}} = \left( \begin{array}{c} 0\\ \varOmega _{\mathrm {MDM}} \\ 0\\ \end{array} \right) \quad \text{ and } \quad {\varvec{\Omega }}_{\mathrm {EDM}} = \left( \begin{array}{c} \eta {{\tilde{\varOmega }}}_{\mathrm {EDM}} \\ 0 \\ 0 \\ \end{array} \right) \end{aligned}$$

with $\varOmega _{\mathrm {MDM}} = - \frac{q}{m} (G B + \left( G-\frac{1}{\gamma ^2-1} \right) \frac{\beta E}{c})$ and ${\tilde{\varOmega }}_{\mathrm {EDM}} = -\frac{q}{2mc} (E + c \beta B)$, $B=|\mathbf {B}|$ and $E=|\mathbf {E}|$. The coordinate system is chosen such that the first component points in radial direction, the second in vertical and the third in longitudinal direction. Note that ${\varvec{\beta }}\times \mathbf {E}$ is anti-parallel to $\mathbf {B}$. This explains the $+$ sign in front of $\left( G-\frac{1}{\gamma ^2-1} \right)$ in the definition of $\varOmega _{\mathrm {MDM}}$ instead of a − sign in Eq. 3.

For the following discussion it is more convenient to write Eq. 2 in matrix form:

7 $$\begin{aligned} \frac{d \mathbf {S}}{dt} = \left( A_{\mathrm {MDM}} + A_{\mathrm {EDM}}\right) \mathbf {S} \end{aligned}$$

with (to simplify the notation we use $\varOmega _{\mathrm {EDM}}$ instead of ${{\tilde{\varOmega }}}_{\mathrm {EDM}}$ from now on)

8 $$\begin{aligned} A_{\mathrm {MDM}}= & {} \left( \begin{array}{ccc} 0 &{} 0 &{} \varOmega _{\mathrm {MDM}} \\ 0 &{} 0 &{} 0 \\ -\varOmega _{\mathrm {MDM}} &{} 0 &{} 0\\ \end{array} \right) \quad \text{ and } \quad A_{\mathrm {EDM}} \nonumber \\= & {} \eta \left( \begin{array}{ccc} 0 &{} 0 &{} 0 \\ 0 &{} 0 &{} \varOmega _{\mathrm {EDM}} \\ 0 &{} -\varOmega _{\mathrm {EDM}} &{} 0 \\ \end{array} \right) . \end{aligned}$$

In the following we assume that the EDM can have a constant term and a time varying component, thus $\eta = \eta _0 + \eta _1 \cos (\omega _a t + \varphi _a)$ as suggested in [4, 11]. The oscillating term is caused by an axion of mass given by the relation $\omega _a = m_a c^2/\hbar$. Assuming $\eta _0, \eta _1 \ll G$, $A_{\mathrm {EDM}}$ in Eq. 7 can be treated as a perturbation.

The solution to first order in $\eta _0$ and $\eta _1$ for arbitrary initial condition of the spin is given in Appendix A. The best sensitivity to $\eta _0$ and $\eta _1$ is obtained by observing a build-up of a vertical polarization of a beam initially polarized in the horizontal plane. Thus we are interested in the behavior of the vertical spin component $S_v(t)$ in the case where the spin points for example initially in the longitudinal direction ($\mathbf {S}(0) = (0,0,1)^T$). Using Eq. 37 in Appendix A one finds:

9 $$\begin{aligned} S_v(t)= & {} \eta _0 \varOmega _{\mathrm {EDM}} \frac{\sin (\varOmega _{\mathrm {MDM}} t)}{ \varOmega _{\mathrm {MDM}}}\nonumber \\&+ \eta _1 \frac{\varOmega _{\mathrm {EDM}}}{2 (\omega _a - \varOmega _{\mathrm {MDM}} ) (\varOmega _{\mathrm {MDM}} + \omega _a)} \left[ -2 \omega _a \sin (\varphi _a) \right. \nonumber \\&\left. + (\omega _a + \varOmega _{\mathrm {MDM}}) \sin \left( (\omega _a - \varOmega _{\mathrm {MDM}}) t +\varphi _a \right) \right. \nonumber \\&\left. + (\omega _{a} - \varOmega _{\mathrm {MDM}}) \sin \left( (\varOmega _{\mathrm {MDM}} + \omega _a) t+ \varphi _a\right) \right] . \end{aligned}$$

We are interested in the behavior close to the resonance condition $\varOmega _{\mathrm {MDM}} \approx \omega _a$. Ignoring in Eq. 9 all fast oscillating terms (i.e. assuming $\varOmega _{\mathrm {MDM}},(\varOmega _{\mathrm {MDM}}+\omega _a) \gg \varOmega _{\mathrm {MDM}}-\omega _a)$ one finds

10 $$\begin{aligned} S_v(t)= & {} \frac{\eta _1 \varOmega _{\mathrm {EDM}}}{2 (\omega _a -\varOmega _{\mathrm {MDM}} ) } \nonumber \\&\times \left( - { \sin (\varphi _a) } + \sin \left( (\omega _a -\varOmega _{\mathrm {MDM}}) t +\varphi _a \right) \right) . \end{aligned}$$

11 $$\begin{aligned}= & {} \eta _1 \frac{\varOmega _{\mathrm {EDM}}}{2\varDelta \omega } \left( -\sin (\varphi _a) + \sin (\varDelta \omega t + \varphi _a)\right) \end{aligned}$$

with $\varDelta \omega = \omega _a - \varOmega _{\mathrm {MDM}}$ For $\varphi _a=0$ this expression coincides with the expression given for NMR experiments [5]. At the resonance, $\omega _a = \varOmega _{\mathrm {MDM}}$, Eq. 11 reduces to

12 $$\begin{aligned} S_v(t) = \frac{\eta _1 \varOmega _{\mathrm {EDM}}}{2} \, \cos (\varphi _a) \, t. \end{aligned}$$

In this case the build-up is linear in time to first order in $\eta _1$.

**Fig. 2:**
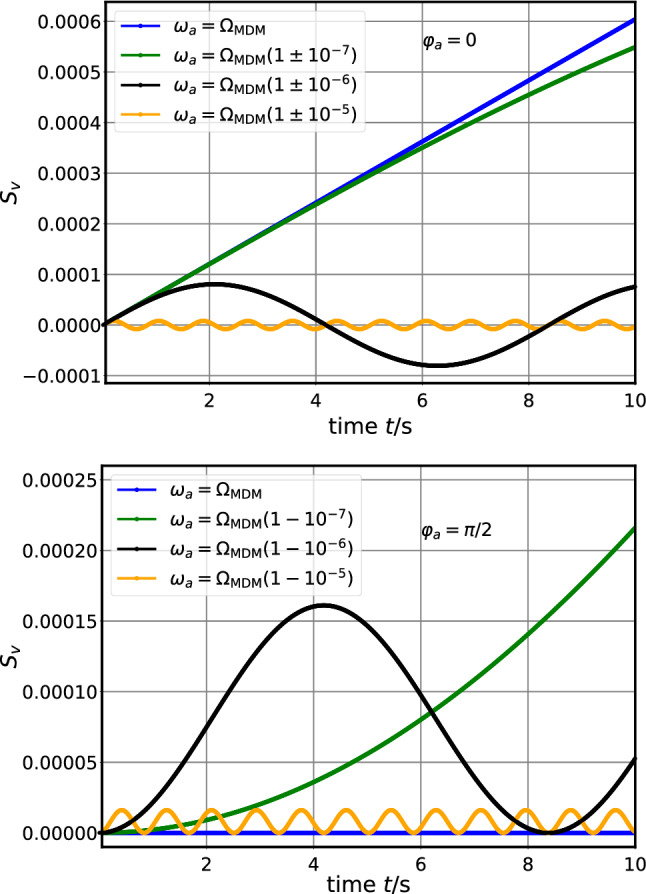
Vertical spin component $S_v$ as a function of time *t* for $\varphi _a=0$ (upper plot) and $\varphi _a=\pi /2$ (lower plot) and for different axion frequencies $\omega _a$ and $\varOmega _{\mathrm {MDM}} = 750{,}000.0 \, {\mathrm{s}^{-1}}$, $\varOmega _{\mathrm {EDM}} \approx 1{,}200{,}000 \, {\mathrm{s}^{-1}}$, $\eta _0=0$, $\eta _1=10^{-10}$

The phase $\varphi _a$ of the axion field is unknown. The experiment should be performed with two bunches in the ring where the polarizations are orthogonal to each other, which corresponds to two phases $\varphi _a$ and $\varphi _a+\pi /2$. This assures not to miss an axion signal. This can also be seen in Fig. 2. It shows the build-up of the vertical spin component $S_v$ as a function of time *t* for $\varphi _a=0$ and $\varphi _a=\pi /2$ and for different axion frequencies $\omega _a$ and $\varOmega _{\mathrm {MDM}} = 750{,}000.0 \, {\mathrm{s}^{-1}}$. This $\varOmega _{\mathrm {MDM}}$ corresponds to typical running conditions with deuterons of $p=970\,{\mathrm{MeV/c}}$ at the COoler SYnchrotron COSY of Forschungszentrum Jülich in Germany. Note that for a given $\varphi _a$ the initial slope is the same independent of $\omega _a$. One clearly observes the resonance behavior. If $\varOmega _{\mathrm {MDM}} = \omega _a$ the polarization build-up is maximal for $\varphi _a=0$. The more $\varOmega _{\mathrm {MDM}}$ deviates from $\omega _a$, the weaker the signal becomes.

For the special case $\omega _a=0$ Eq. 9 becomes

13 $$\begin{aligned} S_v = \frac{\varOmega _{\mathrm {EDM}}}{\varOmega _{\mathrm {MDM}}} \sin (\varOmega _{\mathrm {MDM}} t) \, \left( \eta _0 + \eta _1 \cos (\varphi _a) \right) . \end{aligned}$$

Compared to Eqs. 10 and 12 the signal is two times larger. For the following estimates of statistical uncertainties, we continue to use Eqs. 10 and 12 for conservative results.

## Statistical error estimates

Equations 11 and 12 can now be used to calculate statistical sensitivities under various experimental conditions. We are interested in the error on $\eta _1$.

### Resonance case

The best sensitivity is of course given on resonance, i.e. $\varOmega _{\mathrm {MDM}} = \omega _a$. In this case the spin build-up follows Eq. 12:

14 $$\begin{aligned} S_v(t) = \eta _1 \frac{\varOmega _{\mathrm {EDM}}}{2} \cos (\varphi _a) t. \end{aligned}$$

Assuming that one extracts a beam of *N* particles continuously on a target with the same rate over a time period *T* during which the beam polarization *P* is assumed to be constant and using a polarimeter with an average analyzing power *A* of the scattering process and a fraction *f* of the beam particles detected, the observed vertical polarization (assuming $P_v \ll P$) will be:

15 $$\begin{aligned} P_v(t) = P A S_v(t) = P A \eta _1 \frac{\varOmega _{\mathrm {EDM}}}{2} \cos (\varphi _a) t. \end{aligned}$$

From this polarization measurement $\eta _1$ can be determined with variance

16 $$\begin{aligned} V(\eta _1) = \left( \frac{1}{\varOmega _{\mathrm {EDM}}} \right) ^2 \, \frac{96}{fN (ATP \cos (\varphi _a))^2}. \end{aligned}$$

Details are given in Appendix B.1.

Adding the information from the two bunches with $\varDelta \varphi _a = \pi /2$ one arrives at

17 $$\begin{aligned} V(\eta _1) = \left( \frac{1}{\varOmega _{\mathrm {EDM}}} \right) ^2 \, \frac{96}{fN (ATP)^2}. \end{aligned}$$

**Table 1:** Parameters used for the estimates. The ring radius of the prototype ring is $R=8.9\,{\mathrm{m}}$

		COSY				Prototype ring	
		Proton		Deuteron		Proton	
Momentum	$p/{\mathrm{GeV/c}}$	0.3	3.7	0.3	3.7	0.25	0.30
Spin revolution frequency	$\varOmega _\mathrm {MDM}$/ ${10^6 \, \mathrm{s}^{-1}}$	5.86	72.3	0.233	2.88	7.35	0.0
Axion mass	$m_a$/${\mathrm{eV}}$	$4 \cdot 10^{-9}$	$5\cdot 10^{-8}$	$1.5 \cdot 10^{-10}$	$2 \cdot 10^{-9}$	$5\cdot 10^{-9}$	0
Magnetic field	$B/{\mathrm{T}}$	0.07	0.8	0.07	0.8	0.0	0.033
Electric field	$E/{\mathrm{MV/m}}$	−	−	−	−	7.4	7.4
Stored particles per bunch	*N*	$10^{9}$		$10^{9}$		$10^{10}$	
Fraction detected events	*f*	0.005		0.005		0.005	
Average analyzing power	*A*	0.6		0.6		0.5	
Beam polarization	*P*	0.8		0.8		0.8	
Spin coherence time	$\tau /{\mathrm{s}}$	1000		1000		1000	

### Off-resonance case

For the off-resonance case the vertical polarization is obtained by multiplying Eq. 11 with *PA*:

18 $$\begin{aligned} ~ P_v(t) = \eta _1 P A \frac{\varOmega _{\mathrm {EDM}}}{2\varDelta \omega } \left( -\sin (\varphi _a) + \sin (\varDelta \omega t + \varphi _a)\right) . \end{aligned}$$

In order to determine $\eta _1$, the data have to be fitted to the functional form of Eq. 18. The three fit parameter are $\eta _1$, $\varDelta \omega$ and $\varphi _a$.

**Fig. 3:**
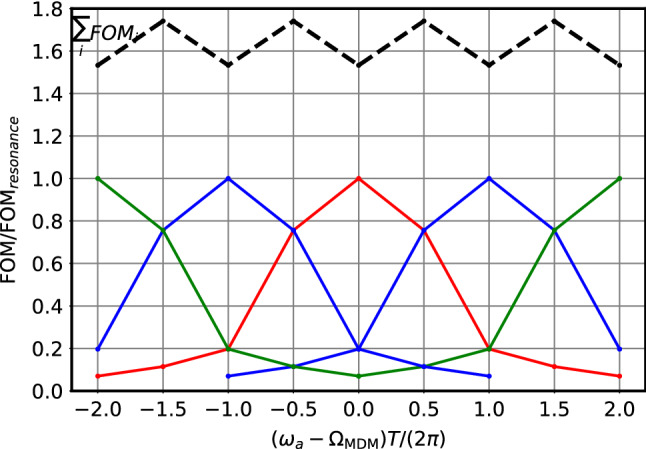
Figure of merit (FOM) as a function of $(\omega _a - \varOmega _{\mathrm {MDM}})T/(2\pi )$ normalized to the FOM at resonance $\varDelta \omega = (\omega _a - \varOmega _{\mathrm {MDM}})=0$. Solid lines: FOM for measurements at $\varDelta \omega T/(2\pi ) = -2,-1,0,1,2$ respectively. Dashed line: sum of FOMs

The central red curve in Fig. 3 shows the figure of merit (FOM) defined as the inverse of the variance of $\eta _1$ as a function of $\varDelta \omega T/(2\pi )$ normalized to the FOM at resonance $\varDelta \omega = \omega _a - \varOmega _{\mathrm {MDM}}=0$ given by the inverse of Eq. 17. If the frequency is off be 1/*T*, with *T* being the measurement duration, the FOM drops to roughly 20%. Details are given in Appendix B.2. This suggests to take measurements separated by 1/*T* in frequency, as indicated by the additional blue and green FOM curves in Fig. 3. The upper dashed black curve which is roughly constant shows the sum of the FOMs from the measurements at the different frequencies. Experimentally one would not run at frequencies $\varDelta \omega T/(2\pi ) = \ldots ,-2 ,-1, 0, 1, 2, \ldots$ as indicated in Fig. 3 but rather sweep the frequency with the speed (= frequency per time) $v=1/T^2$.

To scan a region of $\varDelta f = 1\,{\mathrm{kHz}}$ with a measurement duration of $T=10\,{\mathrm{s}}$ for a single frequency, one would thus need a total measurement time

$$\begin{aligned} \varDelta f T^2 = 10^{5} \, {\mathrm{s}}. \end{aligned}$$

In this frequency range $\eta _1$ would be determined with the same accuracy over the whole frequency range.

### Estimates for the error on the axion-gluon coupling $\frac{C_G}{f_a}$

According to reference [12] the relation between the EDM *d* and $\theta _{QCD}$ is given by $d \approx 10^{-16} \theta _{QCD} e\,{\mathrm{cm}}$. To simplify the discussion we make no distinction between proton and deuteron. $\theta _{QCD}$ is connected to the axion field amplitude $a_0$ and the axion-gluon coupling strength $C_g/f_a$ via $\theta _{QCD} = a_0 \, C_g/f_a$. Using the relation between the axion density $\rho _a$ to the amplitude $a_0 = \sqrt{2 \rho _a}/m_a$ and finally equating $\rho _a$ with the local dark matter density $\rho _{LDM} \approx 0.4\, {{\mathrm{GeV/cm}}^3} \approx 3 \cdot 10^{-42}\, {{\mathrm{GeV}}^4}$ (see reference [13]), assuming that axions saturate the local DM energy, accuracy estimates for $C_g/f_a$ can be obtained as a function of the axion mass $m_a$:

19 $$\begin{aligned} d^{osc.}= & {} 10^{-16} \, \theta _{QCD} \, e\,{\mathrm{cm}} \end{aligned}$$

20 $$\begin{aligned}= & {} 10^{-16} \, a_0 \, \frac{C_G}{f_a} \end{aligned}$$

21 $$\begin{aligned}= & {} 10^{-16} \, \frac{\sqrt{2 \rho _{LDM}}}{m_a} \, \frac{C_G}{f_a} \end{aligned}$$

22 $$\begin{aligned}= & {} 2.5 \cdot 10^{-18} \, \frac{C_G}{f_a} \frac{1}{m_a}\, e{\mathrm{V}} \, {\mathrm{GeV}} \, e {\mathrm{cm}} = \eta _1 \frac{q\hbar }{2mc} \,S. \end{aligned}$$

**Fig. 4:**
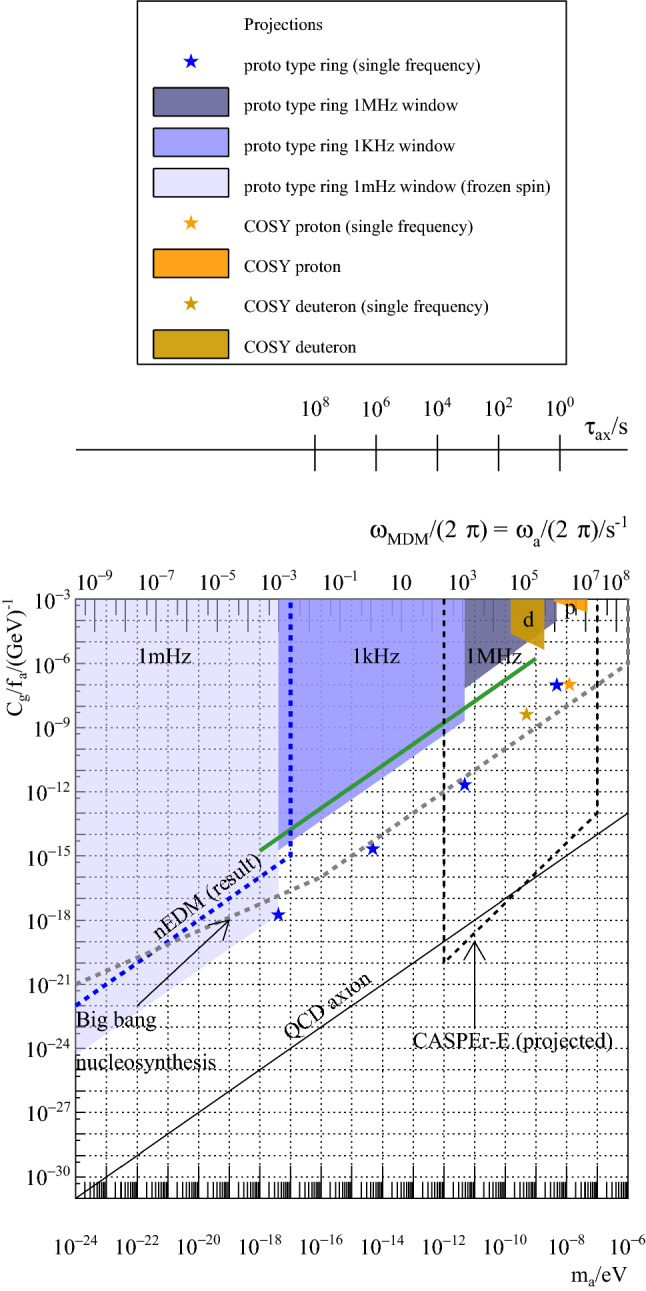
One $\sigma$ limits for the axion-gluon coupling $C_g/f_a$ reachable within 1 year running at a fixed frequency (stars) or over a given frequency range (areas) for COSY (orange) or the prototype ring (blue). In addition, limits reached by the nEDM experiments [15], nucleosynthesis [16] and prospects for NMR experiments [5] are shown schematically. The green line shows the estimates obtained in [1] with simulations

Table 1 gives an overview over frequency ranges accessible at the existing Cooler Synchrotron COSY at Forschungszentrum Jülich in Germany using polarized protons and deuterons and for a planned prototype storage ring with combined electric and magnetic bending fields for an EDM measurement [14]. Other parameters, like number of stored particles *N*, efficiency *f*, analyzing power *A*, polarization *P* and spin coherence time $\tau$ are given as well.

The accuracy estimates are given for two scenarios

1. One year of beam time ($10^7 {\mathrm{s}}$) is spent at a single frequency.
2. In one year of beam time a certain range in frequency is covered.

For the duration of a single measurement, we assure that it does not exceed the axion coherence time, $\tau _{ax}$, given by

$$\begin{aligned} \tau _{ax} =\frac{\pi \hbar }{m_a} Q \end{aligned}$$

with a quality factor $Q=3 \cdot 10^6$ as in reference [1].

The dots in Fig. 4 indicate one-$\sigma$ limits one could reach at COSY running with protons or deuterons and for the prototype ring running at one fixed frequency for one year for each point.

In the second scenario we start with the total running time available in one year, $T_{y} = 10^7\,{\mathrm{s}}$. For the prototype ring, if one wants to span a region of $\varDelta f=1 \, {\mathrm{MHz}}$ in one year, the duration *T* is given by

$$\begin{aligned} T = \sqrt{ \frac{T_y}{\varDelta f} } = 3.2 \,{\mathrm{s}}. \end{aligned}$$

for each frequency interval $\varDelta f_i = {1}/{T}$. For a $1\,{\mathrm{kHz}}$ region, one finds $T=100 \,{\mathrm{s}}$.

The corresponding limits are shown in Fig. 4 as colored areas. The green line shows estimates from reference [1] scaled to match them with the assumptions made in this document about the parameters *N*, *f*, *P*, *A*.

The same is shown for running at COSY. The fact that the limits using a pure magnetic ring are getting worse at smaller frequency is due to the fact that for lower frequencies, the magnetic field is lower, which in turns makes $\varOmega _{\mathrm {EDM}}$ smaller and one loses sensitivity. For the combined ring the electric field is constant, a small magnetic field is added to slow down the spin precession. $\varOmega _{\mathrm {EDM}}$ varies only very little.

## Summary and conclusion

Analytic expressions for the spin motion in presence of an oscillating EDM in storage rings were derived from the Thomas-BMT equation. These were used to give sensitivity estimates for the axion-gluon coupling at COSY and at a prototype EDM ring. This was done for two scenarios: (1) Running at one fixed frequency, (2) covering a wide range in frequency.

The results are in good agreement compared to reference [1] where a numerical approach was used to find sensitivities.

## Data Availability

This manuscript has no associated data or the data will not be deposited. [Authors’ comment: This manuscript has no associated data since it is based on analytic calculations.]

## Appendix A: Solution of equation 7

Equation 7 can be written as

23 $$\begin{aligned} \dot{\mathbf {S}} = \left( A_{\mathrm {MDM}} + \eta {\tilde{A}}_{\mathrm {EDM}}(t)\right) \mathbf {S}. \end{aligned}$$

To solve Eq. 23 we expand the solution in orders of $\eta$

24 $$\begin{aligned} \mathbf {S}(t) = \mathbf {S}_0(t) + \eta \mathbf {S}_1(t). \end{aligned}$$

Entering Eq. 24 in Eq. 23 and keeping only terms up to order one in $\eta$ yields

25 $$\begin{aligned} \dot{\mathbf {S}}_0 + \eta \dot{\mathbf {S}}_1 = A_{\mathrm {MDM}} \mathbf {S}_0 + \eta (A_{\mathrm {MDM}} \mathbf {S}_1 + {\tilde{A}}_{\mathrm {EDM}} \mathbf {S}_0). \end{aligned}$$

Thus

26 $$\begin{aligned} \dot{\mathbf {S}}_0= & {} A_{\mathrm {MDM}} \mathbf {S}_0 , \end{aligned}$$

27 $$\begin{aligned} \dot{\mathbf {S}}_1= & {} \left( A_{\mathrm {MDM}} \mathbf {S}_1 + {\tilde{A}}_{\mathrm {EDM}} \mathbf {S}_0\right) . \end{aligned}$$

Since $A_{\mathrm {MDM}}$ does not depend on *t*, equation 26 has the solution

28 $$\begin{aligned} \mathbf {S}_0(t) = \mathrm {exp}(A_{\mathrm {MDM}} t) \mathbf {S}(0) \end{aligned}$$

with arbitrary initial condition $\mathbf {S}(0)$.

The solution for the Eq. 27 can be found using the variation of constant method:

29 $$\begin{aligned} S_1= & {} \mathrm {exp}(A_{\mathrm {MDM}} t) S(0)\nonumber \\&+ \int _0^t \mathrm {exp}(A_{\mathrm {MDM}}(t-s)) {\tilde{A}}_{\mathrm {EDM}} S_0(t)\mathrm {d}s . \end{aligned}$$

Up to first order in $\eta$ the solution is

30 $$\begin{aligned} \mathbf {S}(t)= & {} \mathbf {S}_0(t) + \eta \mathbf {S}_1(t) \end{aligned}$$

31 $$\begin{aligned}= & {} (1+\eta ) \, \mathrm {exp}(A_{\mathrm {MDM}} t) \, \mathbf {S}(0)\nonumber \\&+ \eta \, \int _0^t \mathrm {exp}(t-s) {\tilde{A}}_{\mathrm {EDM}} \mathrm {exp}(A_{\mathrm {MDM}} t) \, \mathbf {S}(0) \mathrm {d}s \end{aligned}$$

Using Mathematica [17] one finds $\mathbf {S}(t) = A(t) \mathbf {S}(0)$ with

32 $$\begin{aligned} A_{11}= & {} (1+\eta _0) \cos (\varOmega _{\mathrm {MDM}} t) \end{aligned}$$

33 $$\begin{aligned} A_{12}= & {} \frac{\eta _0 \varOmega _{\mathrm {EDM}} (\cos (\varOmega _{\mathrm {MDM}} t)-1)}{\varOmega _{\mathrm {MDM}}} +\,\eta _1 \varOmega _{\mathrm {EDM}} \,\Bigg ( \frac{ (\sin (\varphi _a) (\omega _a \sin (\varOmega _{\mathrm {MDM}}t)- \varOmega _{\mathrm {MDM}} \sin (\omega _a t))}{\omega _a^2-\varOmega _{\mathrm {MDM}}^2} \nonumber \\&+\frac{\varOmega _{\mathrm {MDM}} \cos (\varphi _a) (\cos (\omega _a t)-\cos (\varOmega _{\mathrm {MDM}} t))}{\omega _a^2-\varOmega _{\mathrm {MDM}}^2} \Bigg ) \end{aligned}$$

34 $$\begin{aligned} A_{13}= & {} (1+\eta _0) \sin (\varOmega _{\mathrm {MDM}} t) \end{aligned}$$

35 $$\begin{aligned} A_{21}= & {} \frac{\eta _0 \varOmega _{\mathrm {EDM}} (\cos (\varOmega _{\mathrm {MDM}} t)-1)}{\varOmega _{\mathrm {MDM}}} - \eta _1 \varOmega _{\mathrm {EDM}} \Bigg ( \frac{ ( \cos ((\omega _a -\varOmega _{\mathrm {MDM}}) t+\varphi _a)}{2 (\omega _a-\varOmega _{\mathrm {MDM}}) } \nonumber \\&+\frac{(\varOmega _{\mathrm {MDM}}-\omega _a) \cos ( (\omega _a+\varOmega _{\mathrm {MDM}})t+\varphi _a)-2 \varOmega _{\mathrm {MDM}} \cos (\varphi _a))}{2 (\omega _a-\varOmega _{\mathrm {MDM}}) (\omega _a+\varOmega _{\mathrm {MDM}})} \Bigg ) \end{aligned}$$

36 $$\begin{aligned} A_{22}= & {} 1+\eta _0 \end{aligned}$$

37 $$\begin{aligned} A_{23}= & {} \frac{\eta _0 \varOmega _{\mathrm {EDM}} \sin (\varOmega _{\mathrm {MDM}} t)}{\varOmega _{\mathrm {MDM}}} + \eta _1 \varOmega _{\mathrm {EDM}} \Bigg ( \frac{ ( \sin ((\omega _a -\varOmega _{\mathrm {MDM}}) t+\varphi _a)}{2 (\omega _a-\varOmega _{\mathrm {MDM}}) } \nonumber \\&+ \frac{(\omega _a-\varOmega _{\mathrm {MDM}}) \sin ( (\omega _a+\varOmega _{\mathrm {MDM}})t+\varphi _a)-2 \omega _a \sin (\varphi _a))}{2 (\omega _a-\varOmega _{\mathrm {MDM}}) (\omega _a+\varOmega _{\mathrm {MDM}})} \Bigg ) \end{aligned}$$

38 $$\begin{aligned} A_{31}= & {} -(1+\eta _0) \sin (\varOmega _{\mathrm {MDM}} t) \end{aligned}$$

39 $$\begin{aligned} A_{32}= & {} -\frac{\eta _0 \varOmega _{\mathrm {EDM}} \sin (\varOmega _{\mathrm {MDM}} t)}{\varOmega _{\mathrm {MDM}}} +\eta _1 \varOmega _{\mathrm {EDM}} \, \Bigg ( \frac{ (\omega _a \sin (\varphi _a) \cos (\varOmega _{\mathrm {MDM}} t)-\omega _a \sin (\omega _a t+\varphi _a)}{(\omega _a-\varOmega _{\mathrm {MDM}}) (\omega _a +\varOmega _{\mathrm {MDM}})} \nonumber \\&+\frac{\varOmega _{\mathrm {MDM}} \cos (\varphi _a) \sin (\varOmega _{\mathrm {MDM}} t))}{(\omega _a-\varOmega _{\mathrm {MDM}}) (\omega _a +\varOmega _{\mathrm {MDM}})} \Bigg ) \end{aligned}$$

40 $$\begin{aligned} A_{33}= & {} (1+\eta _0) \cos (\varOmega _{\mathrm {MDM}} t) \end{aligned}$$

Note that $\eta = \eta _0 + \eta _1 \cos (\omega _a t + \varphi _a)$. We are mainly interested in the entries $A_{23}$ and $A_{21}$ which gives the vertical polarization in case of an initial in plane polarization.

## Appendix B: Variance on $\eta _1$

### B.1: Resonance case: variance of a slope

Starting point is Eq. 15

41 $$\begin{aligned} P_v(t) = P A S_v(t) = P A \eta _1 \frac{\varOmega _{\mathrm {EDM}}}{2} \cos (\varphi _a)\, t. \end{aligned}$$

The variance on the slope parameter $s = P A \eta _1 \frac{\varOmega _{\mathrm {EDM}}}{2} \cos (\varphi _a)$ of a straight line is

$$\begin{aligned} V(s) = \frac{\sigma ^2}{N_{\mathrm {points}} V(t)} , \end{aligned}$$

where $\sigma$ is the error on each individual point where the curve is measured. $N_{points}$ is the number of points entering the fit and *V*(*t*) is the variance of the points along the time axis. For evenly distributed values in a time interval *T*, one has $V(t) = T^2/12$. If the polarization is determined from an azimuthal asymmetry one has [18]:

$$\begin{aligned} \sigma ^2 = \frac{2}{n} , \end{aligned}$$

where *n* is the number of events entering the analysis for a single point. Evidently for the total number of events one has $Nf = n N_{points}$.

Putting everything together one finds

42 $$\begin{aligned} V(s) = \frac{24}{fN T^2}. \end{aligned}$$

Translated to the variance on $\eta _1$ one finds the expression given in Eq. 17

43 $$\begin{aligned} V(\eta _1) = \frac{24}{fN (PAT \cos (\varphi _a))^2} \left( \frac{2}{\varOmega _{\mathrm {EDM}}} \right) ^2. \end{aligned}$$

### B.2: Off-resonance case: variance of an amplitude

A polarization given by Eq. 18 leads to the following count rate in the detector:

44 $$\begin{aligned} N(t) \propto 1&+ \eta _1 \frac{PA \varOmega _{\mathrm {EDM}}}{2 \varDelta \omega } \, \left( -\sin (\varphi _a) \right. \nonumber \\&\left. + \sin (\varDelta \omega t +\varphi _a) \right) \cos (\varPhi ) \end{aligned}$$

where $\varPhi$ is the azimuthal angle of the scattered particle. There are three unknowns $\eta _1$, $\varDelta \omega$ and $\varphi _a$. To estimate the uncertainty on $\eta _1$ we consider the extended maximum likelihood method applied to the counting rate in Eq. 44. The log-likelihood function $\ell$ has the form

45 $$\begin{aligned} \ell= & {} \sum _{i=1}^{N_{\mathrm {events}}} \log \left( 1 + \eta _1 \frac{PA \varOmega _{\mathrm {EDM}}}{2 \varDelta \omega } \left( -\sin (\varphi _a) \right. \right. \nonumber \\&\left. \left. + \sin (\varDelta \omega t +\varphi _A) \right) \cos (\varPhi _i) \right) - \log (N_{tot}), \end{aligned}$$

where $N_{tot}$ is the total number of events detected.

To get the covariance matrix for the three unknowns $\eta _1, \varDelta \omega$ and $\varphi _a$ one has to consider the expectation values of the second derivatives of the likelihood function.

The the second derivative with respect to $\eta _1$ it is for example given by

46 $$\begin{aligned} \left\langle \frac{\partial ^2 \ell }{\partial \eta _1^2} \right\rangle = \int _0^T \frac{\partial ^2 \ell }{\partial \eta _1^2} N(t) \mathrm {d}t. \end{aligned}$$

For $\eta _1 /\varDelta \omega \ll 1$ and a measurement time $T=2\pi /\varDelta \omega$ (corresponding roughly to the black curves in Fig. 2), one finds for example for the error on $\eta _1$:

47 $$\begin{aligned} \sigma _{\varphi _a=0}^2= & {} \frac{1}{(\varOmega _{\mathrm {EDM}} ATP)^2 fN} \, \frac{128 \pi ^2 (15 + 2 \pi ^2)}{(3 + 4 \pi ^2)} \nonumber \\\approx & {} \frac{1033}{(\varOmega _{\mathrm {EDM}} ATP)^2 fN} \end{aligned}$$

48 $$\begin{aligned}&\quad \text{ for } \, \, \varphi _a = 0 \nonumber , \\ \sigma _{\varphi _a=\pi /2}^2= & {} \frac{1}{(\varOmega _{\mathrm {EDM}} ATP)^2 fN} \,\frac{128 \pi ^2 (15 - 2 \pi ^2)}{33 - 4 \pi ^2} \nonumber \\\approx & {} \frac{924}{(\varOmega _{\mathrm {EDM}} ATP)^2 fN} \,\nonumber \\&\quad \text{ for } \, \, \varphi _a = \frac{\pi }{2}. \end{aligned}$$

Combining these two measurements leads to a

49 $$\begin{aligned} V(\eta _1) = \frac{488}{(\varOmega _{\mathrm {EDM}} ATP)^2 fN} \ \end{aligned}$$

which is approximately a factor 5 larger compared to the resonance case in Eq. 43.
